# Out-of-pocket expenditure and financial risks associated with treatment of chronic kidney disease in Ethiopia: a prospective cohort costing analysis

**DOI:** 10.1136/bmjgh-2025-019074

**Published:** 2025-06-13

**Authors:** Solomon Tessema Memirie, Mizan Habtemichael, Hamelmal G Hailegiorgis, Leja Hamza Juhar, Tsegay Berhane, Sisay Tesfaye, Workagegnehu Hailu Bilchut, Maekel Belay Woldemariam, Lina Mohammed Ahmedtaha, Ole Frithjof Norheim

**Affiliations:** 1 Addis Center for Ethics and Priority Setting, College of Health Sciences, Addis Ababa University, Addis Ababa, Ethiopia; 2 Bergen Center for Ethics and Priority Setting (BCEPS), Department of Global Public Health and Primary Care, University of Bergen, Bergen, Norway; 3 Department of Internal Medicine, St Paul Hospital’s Millennium Medical College, Addis Ababa, Ethiopia; 4 Department of Internal Medicine, College of Medicine and Health Sciences, Hawassa University, Hawassa, Ethiopia; 5 Department of Internal Medicine, College of Health Sciences, University of Gondar, Gondar, Ethiopia; 6 Department of Internal Medicine, College of Medicine and Public Health, Jimma University, Jimma, Ethiopia; 7 Department of Global Health and Population, Harvard T.H. Chan School of Public Health, Boston, Massachusetts, USA

**Keywords:** Africa South of the Sahara, health economics

## Abstract

**ABSTRACT:**

**Introduction:**

In Ethiopia, most healthcare expenditures are paid out-of-pocket (OOP), while the burden of kidney disease (KD) is rapidly increasing, posing a major public health challenge in low- and middle-income countries, along with a staggering economic burden. We aimed to quantify the extent of OOP health expenditures and the magnitude of associated catastrophic and impoverishing health expenditures (CHE and IHE) for chronic KD (CKD) care in Ethiopia.

**Methods:**

We conducted a prospective costing analysis for CKD care from the patient perspective. We collected data on OOP health expenditures (2023 US$) and household consumption expenditures from a cohort of 433 patients that were followed prospectively for 6 months. Patients were recruited from six health facilities from four constituencies in Ethiopia. We estimated the burden of OOP payments as the sum of direct medical expenditures (DMEs) and direct non-medical expenditures (DNMEs). DMEs were calculated by summing OOP payments for consultations, diagnostic workups, procedures, medications and hospital stays. DNMEs were computed by totalling OOP expenses for transportation, food and lodging. Additionally, we estimated the economic value of productivity losses incurred by patients and/or caregivers due to time spent seeking care. We used descriptive statistics to measure the extent of CHE and IHE. We ran a logistic regression model to assess the drivers of CHE.

**Results:**

The mean annual OOP expenditure was US$2337 (95% CI US$2014 to US$2659) and varied by type of care: US$677 (95% CI US$511 to US$825) for outpatient care, US$2759 (95% CI US$1171 to US$4347) for inpatient care and US$5312 (95% CI US$4644 to US$5919) for haemodialysis. DMEs (particularly haemodialysis) were the major drivers of cost, accounting for 76%–85% of the total OOP expenditure. Transportation expenditures were the major contributors among the DNMEs. Among those who sought outpatient, inpatient and haemodialysis care, 36%, 67% and 90% incurred CHE, respectively, at a 10% threshold of annual consumption expenditures. Among all patients, 25.6% of households were impoverished due to OOP medical expenditures, with the rate substantially higher among those requiring haemodialysis (43.4%). Facility type and the type of visit were significantly associated with the odds of incurring CHE (p<0.05), while adjusting for wealth quintile, disease stage, area of residence (urban/rural), family size, patient age and insurance membership status.

**Conclusions:**

The household economic burden for CKD care is substantial, likely hindering access to necessary treatment and exacerbating the impoverishment, which is prevalent in Ethiopia. This would be an obstacle in achieving universal health coverage and Sustainable Development Goals in Ethiopia.

**WHAT IS ALREADY KNOWN ON THIS TOPIC**

- The burden of chronic kidney disease (CKD) is rapidly rising in low- and middle-income countries such as Ethiopia.
- The extent of out-of-pocket (OOP) health expenditures and the magnitude of associated catastrophic and impoverishing health expenditures for CKD care has not been quantified in Ethiopia and other similar settings in Africa.

**WHAT THIS STUDY ADDS**

- We conducted prospective costing analysis among 433 patients who sought healthcare services for CKD to estimate the extent of OOP medical expenditures and associated impoverishment.
- Patients and their households incur substantial OOP expenditures and associated impoverishing medical expenditures.
- Patients who are receiving haemodialysis are the most likely to be impoverished.

**HOW THIS STUDY MIGHT AFFECT RESEARCH, PRACTICE OR POLICY**

- Expanding access to renal replacement therapy has the potential to protect households from impoverishing medical expenditures.
- In the face of formidable resource constraints in low-income settings such as Ethiopia, prioritising CKD care should be balanced over other most basic health interventions.

## Introduction

 Chronic kidney disease (CKD) is a prevalent disorder and has become a major public health concern globally with an estimated 45 million disability-adjusted life years lost annually.^1^ CKD disproportionately affects low- and middle-income countries (LMICs), where CKD risk factors such as obesity, hypertension and diabetes are rising sharply.^2 3^ In Africa, accurate data on the prevalence of CKD are lacking, but the overall prevalence of CKD in the general population has been estimated at 15.8%, with an associated risk of developing end-stage kidney disease (ESKD).^4^ Globally, lack of access to dialysis services results in 2.3–3.2 million deaths annually.^5^ In 2021, CKD caused nearly 17 000 deaths in Ethiopia.^6^

There are no population-based data on the incidence and prevalence of CKD in Ethiopia. However, risk factors for CKD such as diabetes and hypertension are prevalent.^7^ According to a summary report on the burden of non-communicable diseases (NCDs) in Ethiopia, the prevalence of hypertension and diabetes among the adult population ranged between 16%–27% and 3.2%–8%, respectively.^8^ A meta-analysis of studies in Ethiopia found a CKD prevalence of 35.5% among patients with diabetes.^9^

CKD imposes a substantial financial burden both on patients and the healthcare system.^10^,^13^ Cost of CKD care increases exponentially with each CKD stage progression.^10^ In many African countries, there is little or no access to kidney replacement therapy (KRT: dialysis or transplantation) due to insufficient infrastructure and catastrophic out-of-pocket (OOP) costs and as a result, most patients remain undiagnosed, untreated and ultimately succumb to the disease.^5^ For most patients with ESKD starting dialysis in sub-Saharan Africa, mortality remained very high, largely because of late presentation, frequent dialysis discontinuation and suboptimal dialysis quality.^5^

Reliable health system governance and health financing are vital to meet the health needs of citizens, including those with CKD. A global overview of health systems oversight and financing for kidney care found that only 40%, 63% and 57% of the non-dialysis CKD, dialysis CKD and kidney transplantation, respectively, were publicly funded. In Africa, however, public funding was much less common, with heavy reliance on private funding and substantial OOP expenditures.^14^ OOP expenditures for CKD care are high and exceedingly likely to lead to catastrophic health expenditure (CHE) and impoverishment.^1315^,^17^ In LMICs, kidney diseases are estimated to cause 188 million cases of CHE.^13^

Ethiopia’s healthcare financing system is predominantly characterised by a heavy reliance on OOP payments, particularly for NCD services. Despite efforts to implement pro-poor health financing reforms such as community-based health insurance (CBHI), coverage remains limited, contributing to <1% of total health expenditure (THE) in Ethiopia in 2019/20.^18^ Public health facilities continue to be under-resourced, while private healthcare, although expanding, remains unaffordable for most of the population. The country’s per capita health expenditure stands at US$35, which is below the US$43 average among low-income African countries and significantly lower than the US$86 per capita spending recommended by WHO for the provision of essential health services.^19 20^

The financial burden associated with managing CKD, particularly ESKD requiring dialysis or transplantation, is substantial and largely excluded from existing public health programmes and insurance schemes. Consequently, patients and their families are often forced to shoulder the full cost of care, resulting in CHE and increased risk of impoverishment. Besides, OOP health expenditures are barriers to treatment and a driver of non-adherence and discontinuation, with poorer households worst affected.^21^

Even though CKD care has a long history in Ethiopia, maintenance haemodialysis only began in 2001.^22^ According to a recent study conducted in Addis Ababa (capital city), the expenses for haemodialysis were a substantial economic burden on patients and their households.^23^ However, unlike our study, the previous study did not address the extent of CHE and impoverishing health expenditure (IHE), it was cross-sectional and limited to the capital city and solely on haemodialysis care. Therefore, in this study, we aim to estimate the level of OOP health expenditure, the extent of CHE and IHE and associated factors for CKD care using prospective data collected from several regions across Ethiopia.

## Materials and methods

### Study design

We conducted a prospective costing analysis of care-seeking for CKD from the patient (household) perspective. We collected OOP expenditure data in local currency (Ethiopian birr or ETB) and then converted to US dollars using the median exchange rate for the data collection period (1 January to 31 August 2023), that is, ETB 54.2=US$1.^24^ We also used an exchange rate of ETB 22.2 per unit of purchasing power parity (PPP) US$ (year 2023).^25^

### Study area and population

Ethiopia is home to an estimated number of 126 million inhabitants in 2023.^26^ It is a low-income country, with a 2023 gross domestic product per capita of USUS$1294^27^ and a predominantly (80%) rural population.^28^ Ethiopia is a federal state administratively structured into 12 regions and two city administrations.

The health sector in Ethiopia is a mixed delivery system composed of public and private sector providers.^28^ The public sector owns 73% of the health facilities, while the rest (27%) is owned by private-for-profit and non-for-profit actors.^29^ The public healthcare in Ethiopia is organised in a three-tier system: primary healthcare unit constituting health posts, health centres and primary hospitals serving primarily rural communities; general hospitals (secondary-level) and specialised hospitals (tertiary-level).^28^ The private health sector owns and manages a wide array of health facilities, ranging from primary-level to tertiary-level facilities.^29^

### Study sites and participants recruitment

CKD health services in Ethiopia are available in both public and private-for-profit facilities. Despite the introduction of KRT for acute kidney injury in the early 1980s, maintenance haemodialysis services only became available in 2001.^22^ In the last decade, dialysis services have substantially increased, largely in private-for-profit facilities (24 facilities) and in 11 public facilities.^22 30^ The majority (66%) of the dialysis centres are in Addis Ababa and six regions have at least one dialysis centre, while the rest were without the service.^30^ For patients with ESKD, haemodialysis is the only dialysis modality currently available.^30^

Study participants were patients with CKD receiving treatment at a sample of three public and three private health facilities located in the Addis Ababa, Oromia, Amhara and Sidama regions. The facilities were randomly selected, separately for public and private sectors, from a sampling frame comprising 35 health institutions: 11 public and 24 private-for-profit, where CKD care, including chronic haemodialysis services, is offered. We planned to collect data from a sample of 459 patients assuming a 70% CHE among the poorest quintile and a 20-percentage point difference with the wealthiest quintile and a 2% non-response rate.^16^ Details of the sampling frame and sample size calculation are provided in online supplemental data 1 (sample size and sampling frame).

Patients who were diagnosed with CKD and without malignancies were eligible for inclusion in the study. We included patients fulfilling standard diagnostic criteria, such as albuminuria/proteinuria or haematuria and/or reduced glomerular filtration rate for a duration of >3 months and who are currently receiving ongoing care for CKD. In each facility, all eligible individuals were sequentially recruited from outpatient clinics, dialysis and inpatient units. Subsequently, patients were grouped into three categories based on the characteristics of their initial visits: outpatient (at enrolment, the patient sought outpatient CKD care and has not received dialysis previously), inpatient (admitted for CKD care and has not received dialysis previously) and dialysis (if the patient was enrolled while receiving haemodialysis). We developed these categories since the type of care could be an important driver of cost.

### Data collection

In each facility, eligible individuals were sequentially recruited from outpatient clinics, dialysis and inpatient units over a 2-month period (January–February 2023). We employed face-to-face interviews during exit from the facility using a pretested, standardised questionnaire adapted from a previous study (online supplemental data 2: data collection tool).^31^ We collected data on direct medical expenditure (DMEs) for CKD services such as consultation, diagnostic workup, hospital stays, medication and supplies and direct non-medical expenditures (DNMEs) that include transportation, food and lodging. Furthermore, we asked for the patient’s and/or caregiver’s time losses, how their time would have been spent if she/he was not attending care or taking care of the patient, over-the-counter medication use and the type of health facility visited. During the initial interview, besides data on OOP expenditure, we collected data on the employment statuses of the patient and his/her caregivers’ and if there were any losses in income associated with CKD healthcare visits. Clinical data on CKD stage, comorbidities such as hypertension, diabetes mellitus, etc and medications were obtained from patient records in consultation with the clinician in charge. We collected data on household consumption expenditures (HCEs) by asking the household head, defined as the primary decision-maker or the individual responsible for managing household affairs, for estimates of their expenses on food, rent, utilities (electricity, water, telephone), education, fuel, healthcare and health insurance. Each patient was asked about household financing sources used to cope with OOP expenses.

After the initial interview, the cohort was followed prospectively for 6 months (January–August 2023) through phone calls every 2 weeks to ascertain if there had been any medical visit, the type of visit, services received and related costs incurred. Phone calls were made directly to the patients; however, in cases where the patients failed to respond, we contacted their relatives to ascertain their status (clinical conditions, alive or dead) and healthcare visits.

To facilitate data collection and ensure data quality, we trained four data collectors and a coordinator. The coordinator with the data collectors conducted pilot testing of the Amharic and Oromiffa versions of the questionnaires. Data collection was done using KoboCollect (https://www.kobotoolbox.org/), an open-source tool that enables field workers to gather data using mobile devices or tablets. The platform enabled the coordinator to review the data in real-time and give feedback as appropriate. The data were later cleaned and transferred to Stata software V.14 for analysis.

### Data analysis

We estimated the burden of OOP payments for CKD care over a 12-month reference period, by doubling the sum of DMEs and DNMEs recovered over 6 months. We added up OOP payments for consultation, diagnostic workup, procedures, medications and hospital stay to compute DMEs. Similarly, we added OOP expenditures incurred for transportation, food and lodging to compute DNMEs. Additionally, we estimated an economic value for the productivity losses associated with patients’ and/or caregivers’ time during care seeking. We derived patient’s or caregivers’ time losses by adding the time spent seeking care prior to outpatient consultation, haemodialysis and/or inpatient admission to the duration of outpatient, haemodialysis and/or inpatient visit. We computed productivity losses for income-earning or wage-earning patients and/or caregivers. This was estimated as the sum of reported income loss by the patient plus income losses by caregivers (we accounted for a maximum of three caregivers per patient).

We used the per capita HCE to construct five quintiles accounting for an adult equivalent (AE) score. AE is calculated as follows: AE=(A+αC)^θ^, where A is household adult members, C is the number of children, α is the ‘cost of children’ and θ represents economies of scale. We chose a value of 0.3 for α and 0.9 for θ, because food accounts for a large proportion of total consumption and economies of scale are relatively limited.^32^

We measured the incidence, intensity and distribution of CHE for CKD care by computing OOP expenditures minus any reimbursement from third-party payers divided by annual HCE.^33 34^ CHE were computed at 10% and 40% thresholds (these are the thresholds used by the Sustainable Development Goals (SDGs) to track financial risk protection) of the total HCE and household non-food expenditures (capacity-to-pay, defined as effective income net of subsistence spending), respectively, following the WHO definition of CHE.^35 36^ We calculated catastrophe headcount, weighted headcount, overshoot, weighted overshoot, mean positive gap and concentration indices in accordance with WHO’s methodology and reporting guidelines.^35^ We also conducted a sensitivity analysis using 5% and 25% thresholds of annual consumption expenditure and capacity-to-pay, respectively.

Furthermore, we estimated the proportion of households pushed below the poverty line because of OOP expenditures (poverty headcount), a condition that is usually referred to as IHE. Additionally, we measured the poverty gap—to capture the depth by which households fall below the poverty line—and the normalised poverty gap, which facilitates comparison across countries with varying poverty lines.^35^ We used US$2.15 a day (in 2023 PPP) based on the World Bank Group definition for extreme poverty for low-income countries, which was converted into a poverty line of ETB 17 398 per year.^25^ To estimate the number of households pushed below the poverty line due to OOP expenditures for CKD care, we deducted OOP expenditures from the per capita HCEs (ie, PPP-adjusted amounts in both cases). We also measured the poverty gap as the product of the proportion of the population living in poverty and the average shortfall of the poor from the poverty line. The normalised poverty gap was calculated by dividing the poverty gap by the poverty line. This analysis was conducted for all metrics, both before and after OOP deductions.

Finally, we determined factors associated with CHE, IHE and the extent of OOP expenditure incurred. Initially, we chose potential covariates guided by existing literature and scientific relevance^37 38^ that include income level, residence (urban/rural), facility type (government/private), disease stage, type of initial visit (outpatient/inpatient/dialysis), health insurance coverage, patient age, comorbidities and household size. Each covariate was first assessed in bivariate models, followed by a multivariate analysis for covariates at p value of ≤0.2 in bivariate models. We ran logistic regression to identify the drivers of differences in the odds of CHE and IHE, where ORs were deemed statistically significant at p values of ≤0.05.

The dataset used in this manuscript is available in a publicly accessible repository.^39^

## Results

### Sociodemographic characteristics

Data on OOP expenditures were collected over a period of 6 months from a total of 433 patients (a response rate of 94.3%) with a mean age of 49.6 years and 36% (156) were female. The majority of patients (64%) were recruited from Addis Ababa, where most facilities providing CKD care are located. Seventy per cent patients received care at government facilities. Among them, 55% had ESKD (stage V) and 8% (35 patients, of whom 30 had ESKD) died over the 6-month follow-up period. Nearly 66% (286) patients had health insurance coverage; of these, almost all (99.3%) were covered under the CBHI scheme, while 34% of patients had no insurance coverage (table 1). Common comorbidities and/or complications identified include hypertension, diabetes mellitus and anaemia (table 1). Among patients who were on haemodialysis at enrolment (143), the haemodialysis sessions per week were two to three in 44 (31%) patients, one to two in 65 (45%) patients and less than one in 34 (24%) patients. Despite 237 patients having ESKD, only 177 (75%) had at least one haemodialysis session over the 6 months follow-up period.

**Table 1:** Characteristics of the study participants

Total number of observations	433 (100%)
Total number of cases from health facilities in Addis Ababa	278 (64.1%)
Patient area of residence: urban	261 (93.9%)
Patient area of residence: rural	17 (6.1%)
Outpatient cases	204 (73.4%)
Inpatient	4 (1.4%)
Dialysis	70 (25.2%)
Total number of cases from health facilities in Amhara	70 (16.2%)
Patient area of residence: urban	59 (84.3%)
Patient area of residence: rural	11 (15.7%)
Outpatient cases	39 (55.7%)
Inpatient	13 (18.6%)
Dialysis	18 (25.7%)
Total number of cases from health facilities in Sidama	70 (16.2%)
Patient area of residence: urban	50 (71.4%)
Patient area of residence: rural	20 (28.6%)
Outpatient cases	13 (18.6%)
Inpatient	13 (18.6%)
Dialysis	44 (62.8%)
Total number of cases from health facilities in Oromia	15 (3.5%)
Patient area of residence: urban	12 (80%)
Patient area of residence: rural	3 (20%)
Outpatient cases	4 (26.7%)
Inpatient	0 (0%)
Dialysis	11 (73.3%)
Cases from private facilities	131 (30%)
Mean age in years (95% CI)	49.6 (48.0 to 51.1)
Mean age in years (95% CI): outpatient	54.0 (52.0 to 56.0)
Mean age in years (95% CI): inpatient	46.8 (41.5 to 52.1)
Mean age in years (95% CI): dialysis	42.2 (39.9 to 44.4)
Sex distribution (male)	277 (64%)
Mean number of people per household (95% CI)	6.4 (6.1 to 6.8)
Number (%) of patients with secondary education or more	253 (58.4%)
Number (%) of patients with paid employment	131 (30.3%)
Cases with health insurance membership	286 (66%)
Cases (%) with stage I disease	7 (1.6%)
Cases (%) with stage II disease	16 (3.7%)
Cases (%) with stage III disease	83 (19.2%)
Cases (%) with stage IV disease	89 (20.6%)
Cases (%) with stage V disease (ESKD)	237 (54.7%)
Number (%) of patients with hypertension	365 (85%)
Number (%) of patients with diabetes mellitus	118 (27%)
Number (%) of patients with anaemia	155 (36%)
Number (%) of patients with mineral bone disease	40 (9%)
Number (%) of patients with heart failure	34 (8%)
Number (%) of patients with dyslipidaemia	29 (7%)

ESKD, end-stage kidney disease.

### Magnitude of OOP expenditures

**Figure 1:**
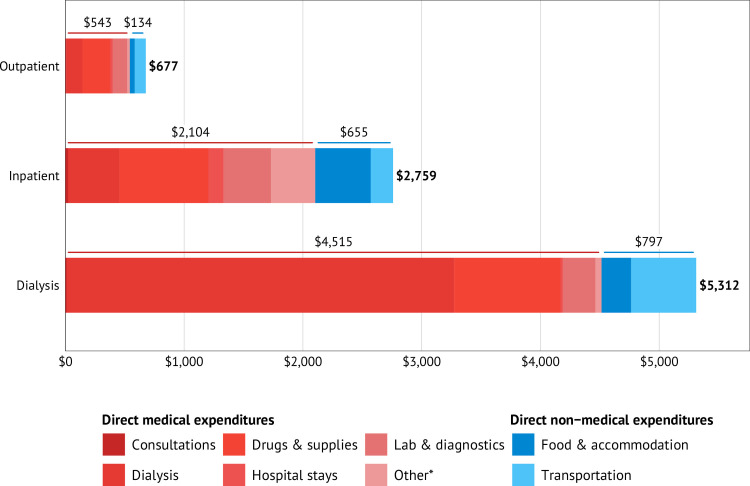
Mean annual out-of-pocket medical expenditures by type of care and expenditures. *Other includes physiotherapy and procedures.

The mean annual OOP medical expenditure for CKD care in 2023 US$ was US$2337 (95% CI US$2014 to US$2659) and varied by type of initial visit from US$677 (95% CI US$511 to US$825) for outpatient department care to US$2759 (95% CI US$1171 to US$4347) for inpatient care and US$5312 (95% CI US$4644 to US$5919) for haemodialysis. DMEs constituted 76%–85% of the total OOP medical expenditure (table 2). Haemodialysis is a major contributor, accounting for 63% of the DMEs followed by medications and supplies (23%). Drugs and supplies accounted for US$235 (95% CI US$179 to US$291), US$748 (95% CI US$308 to US$1189) and US$895 (95% CI US$715 to US$1075) of the outpatient, inpatient and dialysis costs, respectively. Transportation expenses were the largest contributors to DNMEs, accounting for 64% of the DNMEs (figure 1). The total annual OOP medical expenditure also varied by facility type (government/private), disease stage and by health insurance membership status. The mean annual OOP medical expenditures were significantly higher in private facilities (US$5437 vs US$902 in government facilities), in late stages of the disease (US$3818 for stage V vs US$164 for stage I CKD), among patients receiving haemodialysis (US$5,312 vs US$668 for outpatient) and in the richest quintile (US$4618 vs US$497 for the poorest quintile) (table 2). The estimated annual mean (SD) income losses for patients and their caregivers related to CKD care were US$267.1 (US$48.0), which varied by region, residence (urban/rural), facility-type (government/private), disease stage and wealth quintile (details of income losses are provided in online supplemental data 3: ‘income losses and Pen’s Parade’).

**Table 2:** Mean annual OOP medical expenditures (US$) and association with facility type, type of visit, wealth quintile, area of residence, insurance membership and disease stage

Category	Mean (95% CI) expenditure	P value*
Private facilities	5437 (4715 to 6159)	
Government facilities	902 (735 to 1068)	<0.001†
Outpatient	677 (511 to 825)	
Inpatient	2759 (1171 to 4337)	0.007‡
Dialysis	5312 (4673 to 5951)	<0.001‡
Insurance member	1723 (1389 to 2057)	
Not insurance member	3530 (2873 to 4487)	0.351
Residential area: urban	2376 (2029 to 2723)	
Residential area: rural	2040 (1161 to 2919)	0.666
Stage I disease	164 (21 to 307)	
Stage II disease	239 (54 to 421)	0.397
Stage III disease	385 (141 to 629)	0.135
Stage IV disease	785 (409 to 1161)	0.147
Stage V disease	3818 (3326 to 4310)	0.079§
Wealth quintile I (poorest)	497 (339 to 656)	
Wealth quintile II	1728 (1102 to 2354)	0.422
Wealth quintile III	1814 (1163 to 2465)	0.738
Wealth quintile IV	3046 (2301 to 3791)	0.403
Wealth quintile V (richest)	4618 (3698 to 5539)	0.005¶

* P values are based on linear regression analysis using the outcome variable ‘OOP expenditures’ and the independent variables ‘facility type’, ‘type of visit’, ‘insurance membership’, ‘area of residence’, ‘disease stage’ and ‘wealth quintile’. Private facilities, outpatient visits, insurance members, urban residents, stage I disease and the poorest quintile were reference values against which comparisons were made while adjusting for all the independent variables.

† The difference in OOP medical expenditures was significantly higher in private than in government facilities.

‡ The difference in OOP medical expenditures was significantly higher in patients receiving dialysis and inpatient care as compared with outpatient care.

§ The difference in OOP medical expenditures was not significant by disease stage.

¶ The difference in OOP medical expenditures was only significant between the poorest (I) and the richest (V) quintiles.

OOP, out-of-pocket.

#### Cases of CHE and IHE

The annual mean HCE and non-food expenditures were US$5733 (95% CI US$5260 to US$6205) and US$3087 (95% CI US$2703 to US$3471), respectively. There were large variations by wealth quintile, where the richest quintile spent 6.6 times more compared with the poorest quintile (US$12 312 vs US$1865). Food expenditure accounted for 72% and 32% of the total HCEs for the poorest and the richest quintiles, respectively.

For all cases combined (outpatient/inpatient/dialysis), 55.9% and 49.4% of households incurred CHE with a 10% threshold of annual total expenditures and a 40% threshold of annual non-food expenditures, respectively. The CHE estimates were much higher for a lower CHE threshold (5% annual consumption expenditure and 25% non-food expenditure thresholds). The CHE varied by type of visit, where higher CHE incidence (90.2%) was observed for haemodialysis cases. At the 10% threshold of total HCE, the CHE overshoot—representing the average extent to which health payments exceed the threshold—is approximately 37%, ranging from 13% for outpatient care to 75% for dialysis. At the 40% capacity-to-pay threshold, the overshoot is 22.5%, with a range of 12%–41%. The concentration index for the 10% HCE threshold is positive, indicating that CHE is more concentrated among wealthier households, whereas the index for the 40% capacity-to-pay threshold is negative, signifying a greater burden among poorer households (table 3).

**Table 3:** Incidence of CHEs and IHEs by type of visit

	Total	Outpatient	Inpatient	Dialysis
At 10% of annual household consumption expenditures				
CHE headcount (H)	55.9% (242/433)	35.8% (93/260)	66.7% (20/30)	90.2% (129/143)
Concentration indices, H	0.092			
Weighted headcount	58.8%			
Overshoot (O)	35.7%	12.8%	45.4%	75.3%
Concentration indices, O	0.024			
Weighted overshoot	37.9%	14.3%	38.3%	75.5%
Mean positive overshoot	63.9%	35.9%	68.1%	83.4%
At 40% annual non-food expenditure (capacity-to-pay)				
Headcount (H)	50.4% (218/433)	31.9% (83/260)	50% (15/30)	81.1% (116/143)
Concentration indices, H	−0.245			
Weighted headcount	52.5%	36.2%	44.9%	80.6%
Overshoot (O)	22.5%	12.1%	23.8%	41.1%
Concentration indices, O	0.021			
Weighted overshoot	23.7%	12.7%	23.4%	41.1%
Mean positive overshoot	45.7%	38.4%	47.5%	50.6%
CHE incidence at 5% of annual household consumption expenditures and 25% annual non-food expenditure (capacity-to-pay)				
5% threshold	67.9% (294/433)	52.7% (137/260)	73.3% (22/30)	94.4% (135/143)
25% threshold	60.3% (261/433)	43.9% (114/260)	63.3% (19/30)	88.8% (127/143)
IHE incidence and intensity				
Before OOP deductions				
Poverty headcount	5.8% (25/433)	8.5% (22/260)	0	2.1% (4/143)
Poverty gap (US$)	0.04	0.06	0	0.02
Normalised poverty gap	1.9%	2.7%	0	0.7%
After OOP deductions				
Poverty headcount	25.6% (111/433)	15.0% (39/260)	33.3% (10/30)	43.4% (62/143)
Poverty gap (US$)	0.91	0.22	1.46	2.07
Normalised poverty gap	42.5%	10.2%	67.7%	96.1%

CHE, catastrophic health expenditure; IHE, impoverishing health expenditure; OOP, out-of-pocket.

Nearly 6% of study households were below the poverty line (US$2.15 per day, 2023 PPP). This proportion increases significantly to 25.6% after deducting OOP health expenditures from household consumption, representing a >400% rise in the poverty estimate. The rates are considerably higher among patients requiring haemodialysis, reaching 43.4%. The poverty gap also increases dramatically—by a factor of 23—from US$0.04 to US$0.91, with an even greater gap of US$2.07 observed among haemodialysis patients. Similarly, the normalised poverty gap rises from 1.9% to 42.5% when health payments are deducted from household consumption. These findings suggest that the increase in the poverty gap is driven by more households falling below the poverty line and by a worsening of poverty among those already classified as poor (table 3). The Pen’s Parade included as supplementary data (income losses and Pen’s Parade) shows households below the poverty line before OOP medical expenditures and households pushed below the line after incurring OOP expenditures.

The findings from the logistic regressions show that the odds of CHE were statistically significant if the type of visit is for haemodialysis, seeking care in private facilities and marginally significant for disease stage (table 4).

**Table 4:** Factors associated with odds of CHE for CKD

Covariates	Unadjusted		Adjusted		
	OR	95% CI	OR	95% CI	P value
Type of visit					
OPD	1		1		
IPD	3.59	1.61 to 8.00	2.6	1.07 to 6.31	0.035
Dialysis	16.55	9.02 to 30.36	7.61	3.64 to 15.91	<0.001
Wealth quintile					
Q1	1		1		
Q2	1.52	0.83 to 2.76	0.86	0.42 to 1.73	0.667
Q3	1.35	0.74 to 2.46	0.67	0.33 to 1.38	0.275
Q4	2.44	1.33 to 4.50	0.68	0.32 to 1.46	0.324
Q5	3.12	1.67 to 5.83	0.60	0.25 to 1.40	0.237
Disease stage					
1	1		1		
2	2.73	0.26 to 29.07	3.19	0.28 to 36.27	0.350
3	1.90	0.22 to 16.78	2.59	0.28 to 24.25	0.405
4	3.71	0.43 to 32.15	4.54	0.49 to 41.92	0.182
5	19.85	2.34 to 168.48	8.59	0.94 to 78.10	0.056
Facility type					
Government	1		1		
Private	5.06	3.13 to 8.17	2.29	1.26 to 4.15	0.007
Family size	1.05	0.996 to 1.11	1.01	0.94 to 1.09	0.714

CHE, catastrophic health expenditure; CKD, chronic kidney disease; IPD, inpatient department; OPD, outpatient department.

To cope with the high OOP payments, households used various mechanisms. Support from family and/or friends in 178 households (41%), current income in 162 households (37%) and savings in 50 households (12%) were most common. The other coping strategies used included asset sales in 12 households (3%) and borrowing in seven households (about 2%). Most patients (66%) were members of the CBHI scheme; however, only three patients claimed to have received full reimbursement for their CKD care.

## Discussion

This study quantified OOP expenditures, CHE and income losses associated with CKD care in Ethiopia. The mean annual OOP medical expenditures (in 2023 US$) ranged from US$677 for outpatient visits to US$5312 for haemodialysis services. The findings demonstrate that OOP expenditures associated with CKD care can be a considerable economic burden for Ethiopian households. DMEs were major contributors to the total OOP expenditures. Several studies reported similarly very high OOP medical expenditure and DMEs as the drivers of cost but with observable differences with our findings.^23 37 40^ A study from India reported a median total annual cost of US$308 for non-dialysis CKD care that is less than our estimate for outpatient non-dialysis CKD care (US$677).^37^ However, another study from India among patients on haemodialysis reported a monthly cost of US$478 that was comparable to our estimate of US$5320 annually.^40^ The study from Addis Ababa reported a mean annual cost of nearly US$8000 among patients receiving haemodialysis from either public or private facilities.^23^ In this study, all the patients received two to three haemodialysis cycles per week, while in our study, only 31% of the cohort receiving haemodialysis were able to afford two to three dialysis sessions per week and 8.1% of the cohort died during follow-up. The number of dialysis cycles per week is likely to affect the quality of care and survival of patients with CKD. Several studies show that the financial burden of dialysis and non-dialysis CKD care could adversely affect both enrolment and adherence to care.^5 21 30 41^ Furthermore, the study was cross-sectional, unlike ours where we prospectively followed the cohort for 6 months and were able to assess adherence to care and their clinical outcome.

OOP expenditures varied significantly by facility type (government/private), type of care (dialysis/non-dialysis) and household wealth quintile; similar patterns were reported previously.^23 37^ Patients visiting private facilities incurred 6 times more than those visiting government facilities, while dialysis care was 2–8 times costlier than non-dialysis care. Wealthiest households spend nearly 8 times more than the poorest households and are more likely to visit private facilities (90% of patients in the poorest quintile visit government facilities vs 46% for wealthiest quintile). OOP expenditures also vary by insurance membership status (though not statistically significant), where members incur less OOP expenses than non-members. Even though the haemodialysis service is not covered by the CBHI scheme in Ethiopia, partial coverage for medications and supplies for non-dialysis CKD care might have contributed to such a difference.^42^

The risk of CHE at a 10% threshold of annual expenditure was 35.8% for outpatient care, 66.7% for inpatient care and 90.2% for haemodialysis. Comparable figures were observed at the 40% threshold of non-food expenditures (capacity-to-pay). Similarly, 15%, 33.3% and 43.4% of households seeking outpatient, inpatient and haemodialysis services, respectively, would be pushed below the poverty line. OOP expenditures and their associated CHE and IHE for CKD care are high and may be equal to or higher than expenditures for cancer or stroke, as demonstrated by previous studies.^13 15 37^ Patients with advanced disease, visiting private facilities and those requiring haemodialysis were at higher odds of incurring CHE. Even though the difference was not statistically significant, households in the poorest quintile were more likely to incur CHE as compared with the wealthiest quintile, while the wealthy were more likely to use more expensive services in private facilities. CKD disproportionately affects marginalised populations and the financial burden due to OOP expenditure could be an obstacle to access required care, leading to preventable morbidity and mortality.^43^

Households used various coping mechanisms to finance their CKD care expenditures. The most prevalent were receiving support from family and/or friends, current income and savings; however, many households had to sell assets or borrow that might lead to negative long-term economic consequences. Poorer households were more likely to sell assets or borrow as a coping strategy, consistent with previous findings.^44^

Our study was based on a prospective follow-up of patients every 2 weeks, which minimises the risk of recall bias, helps assess patient adherence to care and their clinical status; however, it is not without limitations. Our study was facility-based and only addressed those who sought care at health facilities, which may bias the findings towards wealthier and urban residents. In our sample, the majority were urban residents (88%), primarily from Addis Ababa (the capital city), and only 6% of the households were below the US$2.1 poverty line, substantially lower than the national estimate (27% in 2015 (2017 PPP)).^45^ This may reflect limited access to CKD care among poor and rural populations in Ethiopia. Furthermore, we may have overestimated the expenses for outpatient care, as some patients subsequently received haemodialysis (28 of 260 patients, with a mean dialysis-related expense of US$136) and inpatient care (seven of 260 patients, with a mean expense of US$19). Only 24% of the patients with CKD were in the early stages of the disease (stages 1–3), highlighting the challenge in early diagnosis, which if not treated could progress to ESKD. Delay in the diagnosis of CKD may be a result of low awareness, poor access to appropriate healthcare and associated high cost of care.^46^ We collected HCE data once during the initial interview and did not include it in the biweekly phone follow-ups, as HCE data collection is time-consuming and patients with CKD, particularly those with ESKD, are often unwell. To avoid overburdening participants, we limited the data collection to a single point in time.

Concurrent with large reductions in under 5 mortality and communicable diseases, Ethiopia has seen substantial gains in life expectancy, leading to a steadily rising burden of NCDs.^6 47^ The government of Ethiopia has ratified the United Nations SDGs including SDG target 3.8: to ‘achieve universal health coverage (UHC)’ and SDG target 3.4: to ‘reduce by one-third premature mortality from NCDs’.^48^ The majority of patients with CKD in Ethiopia do not have access to care, particularly to long-term dialysis, and die prematurely.^30^ Dialysis care is unaffordable to most households, OOP becoming an obstacle to care even when services are available. However, dialysis care is also unlikely to be affordable to many low-income countries such as Ethiopia, where healthcare resources are extremely scarce. Among the KRTs, renal transplant is the most cost-effective modality and offers the best prospects for improved survival and quality of life.^49^ Ethiopia in 2015 established the National Kidney Transplant Center with support from Michigan University and has performed >140 kidney transplants.^30 50^ However, the centre has not performed any kidney transplantations since March 2020.^30^

The Ethiopian government spends only US$11 per capita on health and remains highly reliant on OOP payments and external donor support. While a significant portion of health financing comes from donors, these funds are predominantly earmarked for communicable diseases such as HIV, tuberculosis and malaria, as well as maternal and child health services, with limited allocation for NCDs or specialised services like dialysis. OOP payments account for over 30% of THE, a figure that is particularly high for NCD-related services such as CKD care. Health insurance coverage in Ethiopia is also fragmented. The CBHI scheme primarily covers rural and informal sector populations but excludes many high-cost services, including dialysis. Meanwhile, social health insurance for the formal sector has yet to be fully implemented. As a result, the prohibitive cost of dialysis and the limited availability of renal transplantation render these essential services inaccessible to most patients with CKD. For those who do access them, the financial burden is often overwhelming, exposing households to long-term economic hardship and increased vulnerability.^18 51^ In the face of such resource constraints, prioritising CKD care over other most basic interventions that are likely to be more cost-effective would be a formidable challenge in Ethiopia. Building an affordable and sustainable model of care for CKD may require a coordinated global effort and focused attention.^52^

## Conclusions

This study demonstrates that CKD care in Ethiopia imposes a substantial financial burden on households, particularly those requiring haemodialysis and those accessing care from private facilities. OOP expenditures are substantial and contribute to high rates of CHE and impoverishment, with a disproportionate impact on the poorest and most vulnerable populations. Despite the existence of health insurance schemes such as CBHI, coverage remains limited, especially for dialysis services, leading to widespread reliance on coping strategies like borrowing and asset sales. These mechanisms may cause long-term economic hardship, particularly for poorer households. The observed inequalities in access and affordability highlight systemic gaps in health financing and service provision.

Furthermore, the limited availability and affordability of dialysis, coupled with delays in early CKD diagnosis, suggest major barriers to effective CKD management. While Ethiopia has made strides in establishing a transplant centre and improving broader health outcomes, the health system remains severely under-resourced, with low per capita health spending and workforce shortages. Given the rising burden of NCDs in Ethiopia and the country’s commitment to UHC and reduced premature mortality (as outlined in the SDGs), there is an urgent need for a sustainable and equitable model of CKD care. This should include expanding insurance coverage, investing in early diagnosis and prevention, subsidising dialysis and transplant services and integrating CKD management into broader national NCD strategies.

## Supplementary material

10.1136/bmjgh-2025-019074 online supplemental file 1

10.1136/bmjgh-2025-019074 online supplemental file 2

10.1136/bmjgh-2025-019074 online supplemental file 3

## Data Availability

Data are available in a public, open access repository.
