# Supplementary material for: Out-of-pocket expenditure and financial risks associated with treatment of chronic kidney disease in Ethiopia: a prospective cohort costing analysis
Source: BMJ Glob Health. 2025 Jun 13;10(6):e019074. doi: 10.1136/bmjgh-2025-019074 (PMC12164608; doi:10.1136/bmjgh-2025-019074)
Supplement: online supplemental file 1 [file bmjgh-10-6-s001.pdf]

Supplementary data: Sample size and sampling frame

Sample size calculations

We planned to collect data from a sample of 459 patients by assuming 70% catastrophic health expenditure (CHE) among the poorest quintile (Q1), a 20%-point difference with the wealthiest quintile (Q5) and 2% non-response rate using the following formula [1,2]:

$$n = (Z_{\alpha/2} + Z_{\beta})^2 * \frac{p_1(1-p_1) + p_2(1-p_2)}{(p_1 - p_2)^2}$$

Where $Z\alpha/2$ is the critical value of the normal distribution at $\alpha/2$ (i.e., for a confidence level of 95%, α is 0.05, and the critical value is 1.96), Z_β is the critical value of the normal distribution at β (e.g., for a power of 80%, β is 0.2, and the critical value is 0.84), and $p1$ and $p2$ are the expected sample proportions of the two groups.

Sampling frame

CKD care including chronic hemodialysis services are rendered in 35 health facilities: 11 in government facilities and 24 in private-for-profit units [3,4]. We anticipate public and private facilities to have an equal share in the provision of CKD care, therefore, we plan to include three public and three private facilities from the following regions: Addis Ababa, Oromia, Sidama and Amhara. The facilities are selected randomly from these four regions. Facilities that rendered chronic (including those rendering both acute and chronic hemodialysis services) hemodialysis services were included in the actual sampling.

**Addis Ababa City Administration**			
No.	Name of facility	Ownership	Type of hemodialysis (acute or chronic kidney disease)
1	Tikur Anbessa Specialized hospital	Government	Acute
2	St.Paul Hospital's Millennium Medical College	Government	Both
3	Zewditu Memorial Hospital	Government	Chronic
4	St. Peter's TB Specialized Hospital	Government	Acute
5	Minilik Specialized Hospital	Government	Chronic
6	St. Gabriel Hospital	Private	Both
7	Bethel Teaching general Hospital	Private	Both

8	MABD Dialysis Specialty Clinic	Private	Both
9	Sante Medical Center	Private	Both
10	Tom Advanced Renal Care	Private	Chronic
11	Bethzatha General Hospital	Private	Both
12	MCM Korean Hospital	Private	Both
13	Hayat Hospital	Private	Both
14	Tsigereda Dialysis Specialty Clinic	Private	Chronic
15	St. Yared Hospital	Private	Both
16	Girum Hospital	Private	Both
17	Addis Hiwot Hospital	Private	Both
18	Aynalem Primary Hospital	Private	Both
19	Hallelujah General Hospital	Private	Both
20	Ethio-Tebib Hospital	Private	Both
21	Care Land Hospital	Private	Both
22	Ethio-Istanbul	Private	Both
	**Amhara Region**		
No.	Name of facility	Ownership	Type of hemodialysis (acute or chronic kidney disease)
1	Gondar University Hospital	Government	Both
2	Felegehiwot Referral Hospital	Government	Both
3	Woldiya General Hospital	Government	Acute
4	Gambi Hospital	Private	Both
**Oromia Region**			
No.	Name of facility	Ownership	Type of hemodialysis (acute or chronic kidney disease)
1	Jimma University Hospital	Government	Both
2	Adama General Hospital	Private	Both
**Sidama Region**			
No.	Name of facility	Ownership	Type of hemodialysis (acute or chronic kidney disease)
1	Hawassa University Hospital	Government	Both
2	Yanet Specialized Medical Center	Private	Both
**Somali Region**			
1	Sheik Mohammed YaBare	Government	
2			
**Tigray Region**			
1	Ayder Specialized Hospital	Government	Both
**Dire Dawa City Administration Council**			
1	Del Chora	Private	Both
**Harari Region**			
1	Hiwot Fana Hospital	Government	Both

REFERENCES

1. Ramachandran R, Jha V. Kidney Transplantation Is Associated with Catastrophic Out of Pocket Expenditure in India. PLoS ONE. 2013 8(7): e67812. doi:10.1371/journal.pone.0067812

2. Tolla MT, Norheim OF, Verguet S, Bekele A, et al. Out-of-pocket expenditures for prevention and treatment of cardiovascular disease in general and specialised cardiac hospitals in Addis Ababa, Ethiopia: a cross-sectional cohort study. BMJ Glob Health. 2017;2(2):e000280.
3. Muleta MB, Abebe E, Tadesse M, et al. Milestones of renal replacement therapy in Ethiopia. Ethiop Med J. 2020; sup.1:5-13.
4. Mengistu YT and Ejigu AM. Global Dialysis Perspective: Ethiopia. Kidney360;2022:3:1431-1434. doi: https://doi.org/10.34067/KID.0006902021.
