# Supplementary material for: Out-of-pocket expenditure and financial risks associated with treatment of chronic kidney disease in Ethiopia: a prospective cohort costing analysis
Source: BMJ Glob Health. 2025 Jun 13;10(6):e019074. doi: 10.1136/bmjgh-2025-019074 (PMC12164608; doi:10.1136/bmjgh-2025-019074)
Supplement: online supplemental file 2 [file bmjgh-10-6-s002.pdf]

OUT-OF-POCKET HEALTH EXPENDITURE AND ASSOCIATED IMPOVRISHMENT FOR THE TREATMENT OF CHRONIC KIDNEY DISEASE IN ETHIOPIA

Supplementary data: Data collection tool

OUT-OF-POCKET HEALTH EXPENDITURE AND ASSOCIATED IMPOVERISHMENT FOR THE TREATMENT OF CHRONIC KIDNEY DISEASE IN ETHIOPIA

Consent

Hello! My name is ___________. I am representing the Addis Center for Ethics and Priority Setting, Addis Ababa University and University of Bergen. We are conducting a survey regarding the economic impact of accessing care and treatment services for chronic kidney diseases in Ethiopia. As you might be aware, chronic kidney diseases are becoming an increasing burden to the Ethiopian health system. We therefore intend to study the financial risk households face related to accessing health services for care and treatment services for chronic kidney diseases. The evidence generated will inform the process of priority setting for care and treatment services for chronic kidney diseases and facilitate formulation of policy that will address financial risk protection challenges faced by households. For this purpose, we would like to collect information on the direct and indirect out-of-pocket payments patients (households) make to access health services for care and treatment services for chronic kidney diseases in Ethiopia. We are conducting this survey in a sample of public and private hospitals/clinics providing care and treatment services for chronic kidney diseases in Ethiopia. The information you provide in this study will only be used for the purpose stated above.

The information that we collect during this study will be kept confidential. Information about you that will be collected during the research will be put away and no-one but the researchers will be able to see it. You do not have to take part in this research if you do not wish to do so and refusing to participate will not affect your treatment at this hospital or clinic in any way. You will still have all the benefits that you would otherwise have. You may stop participating in the research at any time without losing any of your rights as a patient here. Your treatment will not be affected in any way.

The interview will take about 30 minutes and subsequently we will make a phone call every two weeks to inquire about additional costs related to chronic kidney disease care. We would appreciate to get your consent to be part of this study. We reassure you that the information you provide will be handled anonymously and only for the purpose of the study. Do you agree to be part of this study?

Agree………………….. Disagree……………………….

Thank you for agreeing to be part of this study. If you have any question or if there is anything unclear or if you would like to stop the interview at any point during the course of the interview, please feel free to do so at any time.

OUT-OF-POCKET HEALTH EXPENDITURE AND ASSOCIATED IMPOVRISHMENT FOR THE TREATMENT OF CHRONIC KIDNEY DISEASE IN ETHIOPIA

Should you have any question about the study please contact Dr. Solomon Tessema on +251911403936

To be filled upon completion of outpatient visit or discharge from inpatient admission

Section one: Facility information

1. Region of the heath facility

    1. Amhara
    2. Oromia
    3. Addis Ababa
    4. Sidama

2. Sub city of the heath facility

    1. Addis Ketema
    2. Akaky Kaliti
    3. Arada
    4. Bole
    5. Gullele
    6. Kirkos
    7. Kolfe Keranio
    8. Lideta
    9. Nifas Silk - Lafto
    10. Yeka
    11. Lemi Kura

3. District/Town of the heath facility_____________

4. Facility type

OUT-OF-POCKET HEALTH EXPENDITURE AND ASSOCIATED IMPOVRISHMENT FOR THE TREATMENT OF CHRONIC KIDNEY DISEASE IN ETHIOPIA

1. Government Hospital
2. Private Hospital

5. Health facility name_______

6. Interviewer Name_______

7. Patient’s card number______

8. Diagnosis of the patient (This was used to screen participants)_______

9. Patient Serial number_______

Section two: Socio-demographic characteristics of the patient

In this section, I will ask you about some general information about yourself and the household you live in

1. Age of the patient______

2. What is the patient’s sex?
   1. Male
   2. Female

3 What is the patient's highest educational attainment?
   1. Less than 8
   2. 9 -12
   3. Diploma
   4. Bsc/BA
   5. Msc/MA
   6. Phd
   7. No formal education (can not read)
   8. No formal education (can read)
   9. Other

4. What is the patient’s current occupation?
   1. Government employee

2. Private employee
3. Self-employed
4. Business man/woman
5. Housewife
6. Farmer
7. Student
8. Retired
9. Other

5. If 'Unemployed' in Q4, reason for unemployment

6. Duration of unemployment (recorded in years/months / days)

7. Lost income during the unemployed time (recorded per month or per day

8. What is the patient's marital status?

1. Married
2. Never married
3. Divorced
4. Widow
5. Other

9. Where is the patient's residential address?

1. Addis Ketema
2. Akaky Kaliti
3. Arada
4. Bole
5. Gullele
6. Kirkos
7. Kolfe Keranio
8. Lideta

9. Nifas Silk - Lafto
10. Yeka
11. Lemi Kura

10. District/Town _____________________

11. Sub city

12. Kebele _________________________

13. How is the residential address categorized?

    1. Urban
    2. Rural

14. How many adults live in the household where the patient lives including the patient?

15. How many people live in the household where the patient lives are children ? (< 15)

16. Do you have health insurance ?

    1. Yes
    2. No

17. Type of insurance

    1. Community Based Health Insurance
    2. Other, Specify__________

Section three: Patient medical history

In this section, I will ask you about the care and treatment including medications you receive for your chronic kidney disease.

1. When was the first time you were diagnosed as having chronic kidney disease? ___________

2. Is the stage of chronic kidney disease known?

3. If no, record the most recent creatinine level

4. If yes in , What is the stage of chronic kidney disease?

    1. Stage I
    2. Stage II

3. Stage III A
4. Stage III B
5. Stage IV
6. Stage V (ESRD)

5. Does the patient have any of the following comorbidities or associated conditions?

1. Hypertension
2. Diabetes mellitus
3. Peripheral vascular disease
4. Heart failure
5. Stroke
6. Transient Ischemic Attack
7. Atrial fibrillation
8. Coronary heart disease
9. Anemia
10. Mineral bone disease
11. Dyslipidemia
12. None
13. Other comorbidity , specify

6. Are you currently taking any medication?

7. if yes , list all medications

8. What type of service does the patient receive for the current visit? Multiple response

1. Outpatient care
2. Dialysis session
3. Hospital admission
4. Other type of visit , specify

9. If current visit includes hospital admission, length of stay

10. Do you have a regular follow up outpatient visit with your doctor for the chronic kidney disease?

1. Yes
2. No

11. I f yes, how often?

1. Monthly

2. Quarterly

3. Other, Specify______

12. How many outpatient visits did you have over the past 12 months?

13. During the past 12 months, have you been admitted to a hospital?

14. If yes, how many times were you admitted?

Section four: Out-of-pocket expenditures

In this section, I am going to ask you whether and how much you paid for the health care services you received for the current visit, if you had any, as well as what sources you tapped into to mobilize these funds.

1. For the current healthcare visits, did you spend any amount out-of-pocket for receiving medical services?

    1. Yes

    2. No

2. If no, what was the reason that you did not incur any expenses? Multiple response

    1. Expense covered by CBHI
    2. Expense covered by other type of insurance
    3. The obtained service was exempted
    4. Have a fee waiver
    5. The health facility was near
    6. Other reasons for not incurring expenses

3. Amount of out-of-pocket expenditure for Consultation/registration_____

4. Amount of out-of-pocket expenditure for Laboratory tests/investigation_____

5. Amount of out-of-pocket expenditure for drugs _____

6. Amount of out-of-pocket expenditure for procedures _____

7. Amount of out-of-pocket expenditure for Physiotherapy _____

8. Amount of out-of-pocket expenditure for transportation _____

9. Amount of out-of-pocket expenditure for food costs _____

10. Amount of out-of-pocket expenditure for expenses for care givers _____

11. Amount of out-of-pocket expenditure for other expenses _____

12. How did you cover the expense for the follow up services?

    1. Cost covered by current income of households
    2. Cost covered by own savings
    3. Cost covered by received support from family or friends
    4. Cost covered by Borrowing
    5. Cost covered by selling items
    6. Cost covered by Insurance
    7. Cost covered by equb/Idir
    8. Cost covered by other (amount)

13. For the current dialysis services, did you spend any amount out-of-pocket for receiving medical services?

    1. Yes
    2. No

14. If 'No' , what was the reason that you did not incur any expenses?

    1. expense covered by CBHI
    2. expense covered by other type of insurance
    3. service was exempted
    4. have a fee waiver
    5. the health facility was near
    6. other reasons for not incurring expenses

15. Amount of out-of-pocket expenditure for Consultation/registration_____
16. Amount of out-of-pocket expenditure for Laboratory tests/investigation_____
17. Amount of out-of-pocket expenditure for drugs _____
18. Amount of out-of-pocket expenditure for procedures _____
19. Amount of out-of-pocket expenditure for dialysis sessions _____
20. Amount of out-of-pocket expenditure for hospital beds _____
21. Amount of out-of-pocket expenditure for Physiotherapy _____
22. Amount of out-of-pocket expenditure for transportation _____
23. Amount of out-of-pocket expenditure for food costs _____
24. Amount of out-of-pocket expenditure for expenses for care givers _____
25. Amount of out-of-pocket expenditure for other expenses _____

26. How did you cover the expense for the dialysis service?

1. Cost covered by current income of households
2. Cost covered by own savings
3. Cost covered by received support from family or friends
4. Cost covered by Borrowing
5. Cost covered by selling items
6. Cost covered by Insurance
7. Cost covered by equb/Idir
8. Cost covered by other (amount)

27. When was the first time that you received dialysis?

    27.1 Year of first-time dialysis initiation

    27.2 Number of years since dialysis initiation

28. In what frequency are you receiving dialysis?

    1. Per week
    2. Per two weeks
    3. Per month
    4. Other

29. For the current admission, did you spend any amount out-of-pocket expenditure for receiving medical services? (only for hospital admitted patients)

    1. Yes
    2. No

30. If 'No', what was the reason that you did not incur any expenses?

    1. Expense covered by CBHI
    2. Expense covered by other type of insurance
    3. Service was exempted
    4. Have a fee waiver
    5. The health facility was near
    6. Other reasons for not incurring expenses, Specify

31. Amount of out-of-pocket expenditure for Consultation/registration_____

32. Amount of out-of-pocket expenditure for Laboratory tests/investigation_____

33. Amount of out-of-pocket expenditure for drugs _____

34. Amount of out-of-pocket expenditure for procedures _____
35. Amount of out-of-pocket expenditure for dialysis sessions _____
36. Amount of out-of-pocket expenditure for hospital beds _____
37. Amount of out-of-pocket expenditure for Physiotherapy _____
38. Amount of out-of-pocket expenditure for transportation _____
39. Amount of out-of-pocket expenditure for food costs _____
40. Amount of out-of-pocket expenditure for expenses for care givers _____
41. Amount of out-of-pocket expenditure for other expenses _____

42. How did you cover the expense for the inpatient care?

    1. Cost covered by current income of households
    2. Cost covered by own savings
    3. Cost covered by received support from family or friends
    4. Cost covered by Borrowing
    5. Cost covered by selling items
    6. Cost covered by Insurance
    7. Cost covered by equb/Idir
    8. Cost covered by other (amount)

Section four: Consequences

In this section, I am going to ask you time and wage losses associated with seeking care for your chronic kidney disease.

1. Over the past one month, how much time did you spend or miss from your regular work due to your chronic kidney disease problem or seeking health care for the illness?

(Ask the patient even if they are not formally employed)

--------------Hours

--------------Days

--------------Weeks

2. Do you get paid for the period you missed from work due to illness related to your chronic kidney disease problem or while seeking care? (Ask only for those employed)
    1. Yes, fully……………..

   2. Yes, partially……….
   3. No………………………
3. How many care givers do you have who attend to you on a regular basis?______
4. Over the past one month, how much time did your attendant_1 spend related to your chronic kidney disease problem?

   --------------Hours

   --------------Days

   --------------Weeks

5. What would attendant_1 have done with the time they spent taking care of you?, Read out options, multiple answers are possible
   1. Work (paid)…………………………………...
   2. Work (home and non-paid)……………
   3. Attend school ……………………………….
   4. Leisure…………………………………………..
   5. Spend with family and friends……….
   6. Other (specify)………………………………
6. If the answer is “Work (paid)”, did attendant_1 lose wages while taking care of you?
   1. Yes…………..
   2. No……………
7. If ‘Yes’ , how much wage did attendant_1 lose during the last one-month?

   ……………………………ETB

8. Over the past one month, how much time did your attendant_2 spend related to your chronic kidney disease problem?

   --------------Hours

   --------------Days

   --------------Weeks

9. What would attendant_2 have done with the time they spent taking care of you? , Read out options, multiple answers are possible
   1. Work (paid)…………………………………...
   2. Work (home and non-paid)……………
   3. Attend school ……………………………….
   4. Leisure…………………………………………..
   5. Spend with family and friends……….
   6. Other (specify)………………………………
10. If the answer is “Work (paid)”, did attendant_2 lose wages while taking care of you?
    1. Yes…………..

2. No……………

11. If ‘Yes’, how much wage did attendant_2 lose during the last one-month?

……………………………ETB

12. Over the past one month, how much time did your attendant_3 spend related to your chronic kidney disease problem?

-------------Hours

-------------Days

-------------Weeks

13. What would attendant_3 have done with the time they spent taking care of you? , Read out options, multiple answers are possible
    1. Work (paid)…………………………………...
    2. Work (home and non-paid)……………
    3. Attend school ……………………………….
    4. Leisure………………………………………..
    5. Spend with family and friends……….
    6. Other (specify)………………………………
14. If the answer is “Work (paid)”, did attendant_3 lose wages while taking care of you?
    1. Yes…………..
    2. No……………
15. If ‘Yes’, how much wage did attendant_3 lose during the last one-month?

……………………………ETB

16. If you did not have to come to the hospital to seek care for your chronic kidney disease problem, how would you have used this time? What would you have done? , Read out options, multiple answers are possible
    1. Work (paid)…………………………………...
    2. Work (home and non-paid)……………
    3. Attend school ……………………………….
    4. Leisure………………………………………..
    5. Spend with family and friends……….
    6. Other (specify)………………………………
17. If the answer is “Work (paid)”, did you lose wages
    1. Yes…………..
    2. No……………
18. If ‘Yes’, how much wage did you lose in the last one-month?
    ……………………………ETB

OUT-OF-POCKET HEALTH EXPENDITURE AND ASSOCIATED IMPOVRISHMENT FOR THE TREATMENT OF CHRONIC KIDNEY DISEASE IN ETHIOPIA

Section Five: Household consumption expenditures

In this section, I will ask you about household expenses for essential consumptions including food. Please report all expenses in Ethiopian birr.

On average, how much does your household spend on the following items in a given month or year:

1. How much does your household spend on food and supplies (e.g. raw ingredients, any semi-cooked/cooked/food/snack/sweets etc.) per month ______

2. Does the household consume any home-produced food or goods during the (e.g. wheat, rice, fruits, vegetables, milk, milk products etc.) ______

3. Specify what you produced? ______

4. If you were to buy the same food items from the market, how much would you had to pay on average for an equivalent quantity of what you produced and consumed? ______

5. Does the household received and consumed any food item and supplies as a gift or as an in-kind compensation for any work delivered? ______

6. Please specify what you received?

7. If you were to buy the same food items and supplies (F_1_6) from the market, how much would you have to pay on average for an equivalent quantity of what you received and consumed?

8. How much does your household spend on utilities (electricity, water, telephone) per month

9. How much does your household spend on education (School for children or self) per year

10. How much does your household spend on rent (house/land/shop) per year

11. How much does your household spend on Tobacco, Khat and alcohol per month

12. How much does your household spend on Health care last month

13. How much does your household spend on Health insurance per year

14. How much does your household spend on purchased and replaced household appliances (Utensils ,stove, fridge, food processor, etc.) per year

15. How much does your household spend on Clothes (for the household) per year

16. Maintenance (maintenance and fuel) of bicycle, scooters, Bajaj, cars, household appliances, fridge , stove….per month

17. Reimbursement of loan (for the household use) per month

18. Travel(transportation) costs (for the household) per month

19. Toiletries (soap, detergents…) per month

20. Cosmetics (shampoo, lotion etc.) per month

21. Recreation and entertainment/per month

22. Religious contributions, idder/ per month

23. Rituals, gifts or ceremonies (birthdays, holidays, weddings…) per year

24. Housemaid and guard salary

25. Monthly payment for a condominium

26. Others expenses (amount)

27. Others expenses (specify type)

Section Six: Household income

In this section, I will ask you about the income of the head of the household and all the other income generating and contributing members of the household from official employment, rent or sale of productive assets, and remitence or gifts received on regular basis. Please report all incomes in Ethiopian birr.

1. Total monthly average income of the head of the household (primary income generator) from employment?

2. Total monthly average household income of all the other economically active and contributing members of the household from employment?

3. Total monthly average income of the household from sale or rent of economically productive assets sale or rent of economically productive assets owned by the household?

4. Total monthly average household income from other sources

5. Total monthly average household income from gifts or remittance to members of the household?

6. Total monthly average household income from other sources, specified

Thank you for successfully completing the interview. If you are willing, we would like to contact you every 2 weeks for the next 6months to capture any additional out-of-pocket

payments you make or time lost related to the chronic kidney disease that you have. If you are willing, please provide us with your phone number that we can use to reach out to you or the other care-givers.

Your name _______________________________________ and phone number ________________________

Name of close family member 1 ______________________________ and phone number _________________

Name of close family member 2 _______________________________ and phone number _________________

Time the interview ended_____________________

Signature of the interviewer___________________

Expenses during the follow up period.

Call to be made every 2 weeks for the next 6 months after the first face-to-face interview.

Date of interview________/__________/_________

Call and ask about the condition of the patient and willingness to respond.

1. Status of the patient
    1. Alive and willing to respond ……
    2. Alive and not willing to respond ……….
    3. Passed away ………………….
    4. Unable to reach……………

If the patient passed away or not willing to respond to the interview, thank them for their participation so far and conclude the call.

If the patient is alive and willing to respond to the interview, proceed with the following questions.

The following questions pertain to what happened between the previous interview and now.

1. Did you receive a Dialysis service during the past two weeks?

OUT-OF-POCKET HEALTH EXPENDITURE AND ASSOCIATED IMPOVRISHMENT FOR THE TREATMENT OF CHRONIC KIDNEY DISEASE IN ETHIOPIA

1.Yes

2.No

2. If yes, how many times?___________

3. During the most recent Dialysis service, did you spend any amount of out-of-pocket expenses for receiving medical services?

1.Yes

2.No

4. If the answer is 'Yes', please report how much you spent on each of the following items for the most recent Dialysis service in ETB.(*Consider this dialysis as the more recent health facility visit prior to the current visit) All expenses to be reported in Ethiopian birr*

5. Total expenses

6. Consultation/registration fee

7. Laboratory tests/investigation

8. Drugs

9.Dialysis

10.Procedure

11.Hospital bed day (only for those who had been admitted)

12. Transportation (to and from the health facility)

**For the 'current visit' consider what mode of transport you will be using to return back home.*

13. Extra food cost expenses*(food bought for the child and/or for caregivers who accompanied the child to the health facility)*

14. Additional expenses for care giver *(such as compensation, if any)*

15. Others expenses (amount)

16. Others expenses (describe)

17. During the past two weeks, did you receive any additional medical service other than dialysis?

1. Yes
2. No

19. Other than dialysis, how many encounters/visits did you have in the last two weeks?

20. What type of service did you receive during your visits in the last two weeks?

1. Out patient
2. Refill of medication
3. Procidurre
4. Inpatient
5. Other (describe)________

OUT-OF-POCKET HEALTH EXPENDITURE AND ASSOCIATED IMPOVRISHMENT FOR THE TREATMENT OF CHRONIC KIDNEY DISEASE IN ETHIOPIA

21. Did you receive any new medications in the last two weeks?
    1. Yes
    2. No

22. Did you have any procedures performed in the last two weeks?

    1.Yes

    2.No

23. For each of these encounters/visits, did you spend any amount of out-of-pocket expenses for receiving medical services?

    1.Yes

    2.No

24. If the answer is 'Yes', please report how much you spent on each of the following items for your visits in ETB.
    1. Total expenses ______________
    2. Consultation/registration fee ______________
    3. Laboratory tests/investigation ______________
    4. Drugs ______________
    5. Procedure ______________
    6. Hospital bed day *(only for those who had been admitted)* ______________
    7. Transportation *(to and from the health facility) *For the 'current visit' consider what mode of transport you will be using to return back home.* ______________
    8. Extra food cost expenses*(food bought for the child and/or for caregivers who accompanied the child to the health facility)* ______________
    9. Additional expenses for care giver *(such as compensation, if any)* ______________
    10. Others expenses (amount) ______________
    11. Others expenses (describe) ______________

25. Overall, how much money have you spent to get healthcare for receiving medical services in the last two weeks?(*Some households may not remember all the details above)

Thank you again for completing the follow up phone call interview successfully. Please provide any remark or feedback you may have related to the study.
