# Supplementary material for: Out-of-pocket expenditure and financial risks associated with treatment of chronic kidney disease in Ethiopia: a prospective cohort costing analysis
Source: BMJ Glob Health. 2025 Jun 13;10(6):e019074. doi: 10.1136/bmjgh-2025-019074 (PMC12164608; doi:10.1136/bmjgh-2025-019074)
Supplement: online supplemental file 3 [file bmjgh-10-6-s003.docx]

**Supplementary data: Income losses and Pen parade**

Estimated mean income losses of CKD patients and their caregivers in 2023 USD by region, facility type, disease stage, residential category and wealth quintile.

| Category and No. of observations | Mean (SD) income loss ($) |
| --- | --- |
| All cases (433) | 267 (998) |
| Amhara region | 52 (279) |
| Oromia region | 6 (23) |
| Addis Ababa city administration | 388 (1,212) |
| Sidama region | 60 (315) |
| Private facilities (137) | 345 (102) |
| Government facilities (296) | 231 (52) |
| Residential area: urban (382) | 273 (1,035) |
| Residential area: rural (51) | 221 (667) |
| Stage I disease (7) | 47 (126) |
| Stage II disease (16) | 221 (626) |
| Stage III disease (83) | 70 (263) |
| Stage IV disease (89) | 455 (1,416) |
| Stage V disease (237) | 277 (1,004) |
| Wealth quintile I (poorest) (87) | 108 (526) |
| Wealth quintile II (87) | 233 (875) |
| Wealth quintile III (86) | 420 (1,363) |
| Wealth quintile IV (87) | 355 (1,143) |
| Wealth quintile V (richest) (86) | 241 (879) |


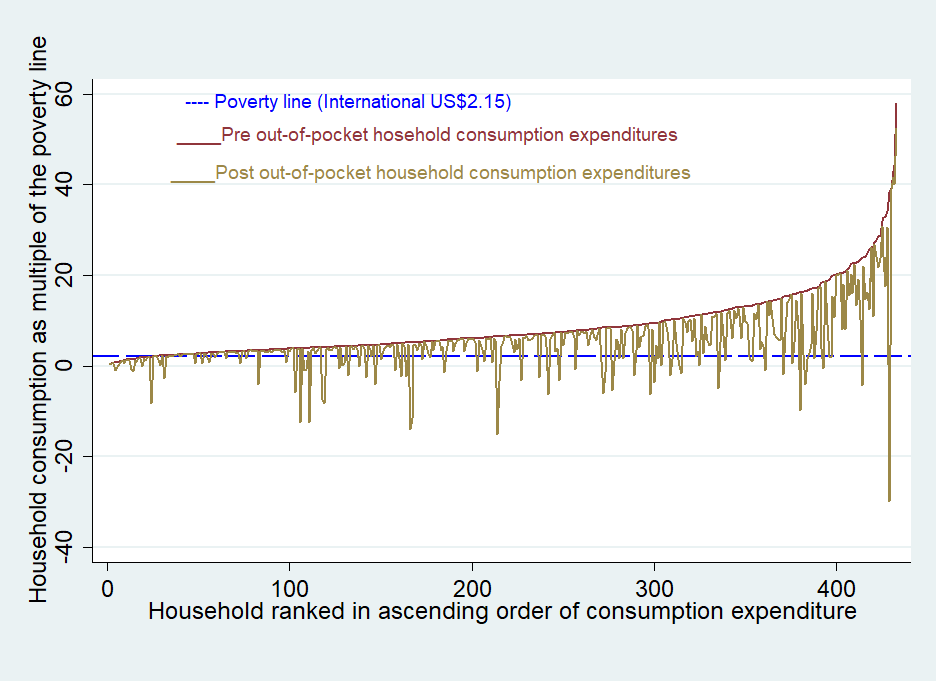


Impact of out-of-pocket expenditures for chronic kidney disease care on annual household consumption expenditures
